# Immunohistochemical expression of Drosha is reduced in eutopic and ectopic endometrium of women with adenomyosis

**DOI:** 10.1590/1414-431X2022e12375

**Published:** 2022-12-12

**Authors:** I. Ormenezi, A. Ribeiro-Silva, J.C. Rosa-e-Silva, J. Meola, F.J. Candido-dos-Reis, O.B. Poli-Neto

**Affiliations:** 1 Departamento de Ginecologia e Obstetrícia, Faculdade de Medicina de Ribeirão Preto, Universidade de São Paulo, Ribeirão Preto, SP, Brasil; 2 Departamento de Patologia, Faculdade de Medicina de Ribeirão Preto, Universidade de São Paulo, Ribeirão Preto, SP, Brasil

**Keywords:** Adenomyosis, Pelvic pain, Drosha, Immunohistochemistry, miRNA, Bleeding

## Abstract

The objective of this study was to evaluate the immunohistochemical expression of Dicer, Drosha, and Exportin-5 in the eutopic and ectopic endometrium of women with adenomyosis. Twenty-two paired ectopic and eutopic endometrium from women with adenomyosis and 10 eutopic endometrium samples from control women undergoing hysterectomy were included in the study. Paraffin-embedded tissue blocks were cut and stained for immunohistochemistry. The percentage of epithelial cells positively marked was identified digitally after an automated slide scanning process. Mann-Whitney test or Wilcoxon signed-rank test was performed for independent and paired groups, respectively. A lower expression of Drosha was observed in the eutopic endometrium of women with adenomyosis than in the eutopic endometrium of women without the disease (69.9±3.4% *vs* 85.2±2.9%, respectively) (P=0.016; 95%CI: 3.4 to 27.4%). We also detected lower Drosha expression in the ectopic endometrium of women with adenomyosis than in the eutopic endometrium of the same women (59.6±3.2% *vs* 69.9±3.4%, respectively) (P=0.004; 95%CI: 2.3 to 16.7%). Additionally, we observed a correlation between Drosha expression in the ectopic and paired eutopic endometrium (P=0.034, rho=0.454). No significant difference in Dicer or Exportin expression was observed. Predominant pattern of cytoplasmic staining for the anti-Drosha antibody and both a nuclear and cytoplasmic pattern for the anti-Exportin antibody were observed. Drosha expression was significantly lower in the endometrium of women with adenomyosis compared to the eutopic endometrium of asymptomatic women without the disease. Furthermore, its expression was lower in the ectopic endometrium but correlated to the paired eutopic endometrium.

## Introduction

Adenomyosis is a gynecological condition characterized by the presence of glandular and stromal endometrial tissue within the uterine myometrium. It was first described in the mid-19th century, and the estimated prevalence ranges from 10% to 30% in women of reproductive age (1). At least two factors are responsible for the inaccuracy in identifying the disease. First, although it is associated with pain (painful menses and cyclic pain), and/or heavy menstrual bleeding, and/or infertility, about a third of women with adenomyosis are asymptomatic (2). Therefore, symptoms are not good predictors of the disease. A second but not less important point is the lack of uniformity in histological criteria for diagnosing the disease (3). The risk factors, in turn, are not yet fully understood. Classically, it has been described as a condition that affects multiparous women between the fourth and fifth decade of life and those undergoing uterine surgeries such as curettage, cesarean section, and myomectomy (4). It is also possible that there is some genetic susceptibility to the disease (5).

The gold standard for diagnosis is a histological analysis of the material obtained by biopsy or hysterectomy. Preoperative diagnosis is a challenge (6). Recently, standardization of ultrasound findings reporting, particularly with the consensus of Morphological Uterus Sonographic Assessment (MUSA), seems to be contributing to this, including a proposal for morphological classification and extension of the disease (7). Like ultrasound, magnetic resonance imaging has also emerged as a promising noninvasive tool in the presumed diagnosis of the disease (8). Both methods appear to have good accuracy (9), although this needs confirmation in future studies. Treatment protocols for adenomyosis are not uniform (10). Hysterectomy is still often used for definitive treatment of the disease; however, there is already evidence that progestin use is effective in controlling pain and abnormal uterine bleeding associated with the condition (11).

Mechanisms involved in the etiology of adenomyosis are not fully elucidated and there are two more widely accepted theories: the invagination theory and the metaplasia theory (12). The first points to an invagination of endometrial cells into the myometrium through the junctional zone, based on the tissue injury and repair (TIAR) hypothesis, which has been remodeled by the endometrial-myometrial interface disruption (EMID) theory. The metaplasia theory proposes that Müllerian remnants or stem cells present in the myometrium undergo inadequate differentiation, leading to the development of ectopic endometrial tissue. From a pathophysiological point of view, numerous processes appear to be involved in a delicately orchestrated and self-sustained cycle, including cell damage, proliferation, fibrosis, inflammation, tissue hypoxia, neuroangiogenesis, increased estrogen production, and uterine hyperperistalsis (13).

Since the end of the 20th century, more specifically from the early 21st century forward, the development of genomic science, together with the considerable increase in computational processing capacity, has allowed us to improve our understanding of the biology of diseases in a way never seen before. Significant alterations in the expression of genes associated with adenomyosis have been identified (14,15), but the determinants of modulation of these transcripts are still an enigma. Within this context, microRNAs (miRNAs) have emerged as key post-transcriptional regulators in the control of several physiological processes, including the endometrium and various disorders such as endometriosis, abnormal uterine bleeding, and endometrial cancer (16). miRNAs are small noncoding RNA molecules that contain about 22 nucleotides. They are well conserved from an evolutionary point of view, and their major function is the post-transcriptional gene regulation of at least 60% of human genes. Briefly, they are transcribed by RNA polymerase II as primary miRNA (pri-miRNA), which is cleaved still in the nucleus by the Drosha enzyme, forming the precursor miRNA (pre-miRNA), which is transported to the cytoplasm by Exportin-5, where it is processed by Dicer, an endonuclease (RNase III), into mature miRNA (17). miRNAs modulate the post-transcriptional process by directly interacting with mRNA through complementary base pairing. In general, this interaction culminates in mRNA silencing through translational repression or mRNA degradation; although less frequently, they promote transcriptional activation and translational enhancement (18).

Guo et al. (19) observed 156 miRNAs differentially expressed between normal eutopic endometrium and eutopic and ectopic endometrium of women with adenomyosis. Among the top 10, some were upregulated (miR-143, miR-532-3p, miR513a, miR-466, and miR-451a) and others were downregulated (miR-10b, miR-371b-5p, miR92b- 5p, miR-30c, and miR-100). Additionally, they showed that miR-10b acts directly on the ZEB1 and PIK3CA genes in endometriotic epithelial cells to modulate tissue invasiveness via E-cadherin upregulation and Akt phosphorylation inhibition. Another miRNA associated with the development of adenomyosis appears to be miR-17. It is upregulated in endometrial tissue from patients with adenomyosis and is associated with lower PTEN expression, suggesting that miR-17 may be one of those responsible for inhibiting endometrial cell apoptosis (20). In addition to these changes, it has been observed that the proliferation of adenomyotic smooth muscle cells promoted by Lin28B overexpression may be caused by the downregulation of miR-Let-7a in the junctional zone of smooth muscle cells of patients with adenomyosis (21). Furthermore, it has recently been suggested that reciprocally dysregulated expression of miR-10b, miR-200c, and miR-191 could be useful as a low-invasive method of adenomyosis diagnosis (22). Interestingly, most of these miRNAs are somehow associated with endometrial cancers (23), as are proteins involved in the biogenesis of these molecules (24).

This pilot study aims to evaluate the immunohistochemical expression of the Dicer, Drosha, and Exportin-5 proteins in the eutopic and ectopic endometrium of women with adenomyosis.

## Material and Methods

### Study design

This study had a retrospective study design based on prospective series. Clinical data was retrieved from medical records previously collected between 2010 and 2017. Ectopic and eutopic endometrium samples from women with adenomyosis and eutopic endometrium samples from women undergoing hysterectomy (without evidence of adenomyosis, leiomyomatosis, or endometriosis) were included in this study. All samples were arranged in paraffin blocks. The study was previously approved by the Institution’s Research Ethics Committee and the National Research Ethics Committee (process number 79643617.8.0000.5440).

The material was identified in the pathology service (SERPAT) of the Clinical Hospital of the University of São Paulo at Ribeirão Preto Medical School (HCFMRP-USP). The corresponding blocks were consecutively revised in descending chronological order to check the eligibility criteria and the technical requirements of quality and representativeness of the target tissue (eutopic and ectopic endometrium). This work was developed in the Oncopathology Laboratory of the Department of Pathology at the same institution. All blocks were reviewed by three authors (OBPN, ARS, and IO) and included only after consensus.

### Eligibility criteria

Samples of women with diffuse adenomyosis, who were non-nulliparous, had pelvic pain, and had abnormal uterine bleeding that did not respond to clinical treatment with combined hormonal contraceptives or isolated progestogens, without associated pathologies (leiomyomatosis, endometriosis, endometrial polyp, and/or pelvic inflammatory disease), not users of any intrauterine device, and who underwent total hysterectomy after a minimum period of two months without the use of hormonal drugs were included in the study. As controls, samples of non-nulliparous women who underwent total hysterectomy and salpingo-oophorectomy for the treatment of benign ovarian epithelial tumors without a history of abnormal uterine bleeding or pelvic pain or use of hormonal contraceptives in the past two months were selected. We performed a preselection of samples that inferred the phase of the menstrual cycle on the day of the last menstruation and included only those with histological findings compatible with the proliferative phase of the menstrual cycle. We considered the following morphological characteristics: straight and narrow glands, and cuboidal glandular epithelium, eventually with pseudostratification; dispersed nuclear chromatin and mitotic figures present; stromal cells also with mitotic activity and ill-defined borders. Twenty-two paired samples of eutopic and ectopic endometrium from women with adenomyosis and 10 samples of eutopic endometrium from control women were included. None of the selected participants were menopausal.

### Clinical information

The care team prospectively extracted and recorded the clinical information from the medical records. In the included cases, there were no missing data.

### Immunohistochemistry

We performed three consecutive serial sections whenever possible, using one for each antibody. For each antibody of interest, all preparations were performed together in a single analysis. After immunostaining, slides were randomly assigned to observers who were blinded to clinical information. As adenomyosis is a typical lesion, it was not possible to blind the observers to this information. However, to represent the eutopic endometrium, we selected sections without an endometrial component in the myometrium, so it was not possible to define which section was from control and which was representative of women with adenomyosis. The antibodies used are listed in Table 1.

**Table 1:** Description of antibodies used in the study.

Antibodies	Brand	Clone	Dilution	Positive control
Anti-Drosha	ABCAM/UK	ab102015	1:100	Lung adenocarcinoma
Anti-Dicer	ABCAM/UK	ab82539	1:60	Placenta
Anti-Exportina-5	ABCAM/UK	ab129006	1:200	Colon

No primary antibody was used for the negative control.

Formalin-fixed, paraffin-embedded tissue blocks were cut in a microtome (LEICA^®^ RM2245, Germany) into 4 µm-thick sections and deposited on previously treated glass slides (ENTELLAN^®^, Germany) to perform immunohistochemical reactions. The material was submitted to deparaffinization in xylene three times for five minutes and then subjected to dehydration in an alcohol gradient (absolute I, absolute II, 95, 90, 80, and 70%) for one minute each. Hydration was carried out in running water and distilled water. Subsequently, antigen recovery was performed by boiling the slices in 0.1 M citrate buffer pH 6.0 for 40 min in a steam pan (90°C), except for anti-Dicer antibody, for which antigen recovery was performed using 10× DIVA Decloaker buffer (USA). After recovery, the slides were left at room temperature for 20 min and then blocked endogenous peroxidase with a commercial kit (DHP-125, Spring Bioscience, USA). Later, TBST (TRIS-buffered saline 0.05 with Tween 20) was used to wash the slides for five minutes. To block nonspecific connections, the ULTRAV kit (Biogen, USA) was used for 10 min. Then, incubation was performed with the primary anti-human antibody diluted in 0.1% BSA (bovine serum albumin) overnight at -4°C.

After incubation with the primary antibody, washing was performed with TBST with the addition of REVEAL Complement (DCMT-15, Spring Bioscience) - secondary antibody - for 10 min. The material was then incubated for 15 min in horseradish peroxidase conjugate (DHRR-125, Spring Bioscience). The reaction was developed with DAB chromogen according to manufacturer's specifications, (Spring Bioscience). The slides were washed in distilled water for five minutes and counterstained with Harris hematoxylin for 40 s. The sections were then hydrated in increasing alcohol gradients, diaphonized in xylol, and mounted in a permanent medium.

### Histological image analysis

Slide selection was based on the highest representation of typical endometrial epithelial tissue (eutopic and ectopic) by consensus of two observers (IO, ARS). To assess immunostaining, a conventional light microscope (Axiostar Plus^®^, Zeiss, Germany) was used and all fields containing the lesions were examined, with cells stained dark brown after the reactions being considered positive. On each slide, 10 fields of 40× magnification were analyzed, prioritizing the most intensely marked fields. Each slide was independently classified by two authors (IO, ARS) with respect to the percentage of positive cells in 0 (0-5%), + (6-25%), ++ (26-50%), and +++ (51%-100%) to check the agreement. In case of disagreement, a third author was consulted, and an agreement was reached. A consensus was obtained after reclassifying the slides as predominantly ‘negative marking' (categories 0 and +) or ‘positive marking' (categories ++ and +++).

For quantitative analysis of the marking, we used the QuPath digital tool (https://qupath.github.io) (25). For this, we proceeded with the automated slide scanning process using the Olympus BX61VS slide scanner system (Olympus Optical do Brasil Ltda, Brazil) through the VS120 Virtual Slide Microscope software (Olympus Optical do Brasil Ltda), located in the Multi-user Laboratory of Electronic Microscopy (LMME) of the Department of Cell Biology and Pathogenic Bioagents of FMRP. Five areas of adenomyotic lesions and two areas of eutopic endometrium were scanned for each slide using a 20× magnification objective. All images were saved in tiff format.

### Digital image analysis

The obtained images were loaded as bright-field type, suitable for the DAB staining we used. This step is imperative and interferes with the automated marking separation process. It uses a color deconvolution method within the RGB (red, green, and blue) spectrum. Once loaded, the images were annotated. This annotation process represented the selection of objects or areas of interest for analysis. Although this process could be automated, we preferred to manually select all the glandular tissue represented in the image. For this, we used two available drawing tools: polygon and brush. With the former, a region can be delimited using fine polygonal lines, and with the latter, a sector can be selected using a brush of varying thickness.

After the selection of areas of interest, we proceeded with the cell detection process using a built-in function called positive cell detection, as it was the one that best suited our preliminary analyses. The parameters referring to the detection of nuclei, cells, and background intensity were, as a rule, standardized between the images, using the default settings suggested by the software. Fine individual adjustments were made in the cell boundary detection parameter due to a morphological variation in cell size between slides. The ratio between positive cell count (independent of intensity) and total cell count was used to generate the cell marking index, reported as a percentage.

### Statistical analysis

The antibody immunostaining quantification data are reported as the mean and standard error of percent positive cells among total number of cells identified per sample. To analyze the difference in percent number of epithelial cells positively marked by antibodies between the eutopic endometrium of women without adenomyosis and the eutopic endometrium of women with adenomyosis, the Mann-Whitney test for two independent groups was performed. To analyze the difference in percent of epithelial cells positively marked by antibodies between eutopic endometrium and ectopic endometrium of women with adenomyosis, we used the Wilcoxon test for two paired samples (Wilcoxon signed-rank test). To analyze the correlation between the percent of epithelial cells positively marked by antibodies between the eutopic endometrium and the ectopic endometrium of women with adenomyosis, Spearman's correlation method was applied. A P-value less than 5% was established as significant in all statistical analyses performed.

## Results

Table 2 shows the characteristics of the included patients. Table 3 shows the mean and standard error (SE) of the percentage of positive epithelial cells, as well as the absolute number of cells examined for each condition.

**Table 2:** Clinical characterization of patients included in the study.

	Cases (n=22)	Controls (n=10)	P value
Age, mean±SD	41.8±3.6	42.2±3.1	0.751
Menarche, mean±SD	11.9±1.7	12.6±1.1	0.258
Pregnancies, median (range)	4 (1-8)	2.5 (2-4)	0.059
Parity, median (range)	2.5 (1-4)	2 (1-4)	0.261
Location, n (%)		-	-
Anterior wall	6 (27.3)	-	-
Posterior wall	8 (36.4)	-	-
Mixed	8 (36.4)		
Uterine layer involvement, n (%)		-	-
Type 1-2	15 (68.2)	-	-
Type 2-3	5 (22.7)	-	-
Type 1-2-3	2 (9.1)	-	-

Type1: junctional zone; type 2: middle myometrium; type 3: outer myometrium. *t*-test or Mann-Whitney test.

**Table 3:** Percentage of positive epithelial cells for each antibody in each condition.

Antibodies/Condition	Endometrium	% (mean±SE)	Cells counted
Anti-Drosha			
Control	Eutopic	85.2±2.9	3168
Adenomyosis	Eutopic	69.9±3.4	3818
	Ectopic	59.6±3.2	2562
Anti-Dicer			
Control	Eutopic	2.4±1.8	2932
Adenomyosis	Eutopic	0.1±0.0	3015
	Ectopic	0.2±0.0	2232
Anti-Exportina-5			
Control	Eutopic	67.8±7.6	3352
Adenomyosis	Eutopic	76.3±2.6	3190
	Ectopic	72.4±3.4	2436

A lower expression of Drosha was observed in the eutopic endometrium of women with adenomyosis than in the eutopic endometrium of women without the disease (69.9±3.4% *vs* 85.2±2.9%, respectively) (P=0.016; 95%CI of the difference: 3.4 to 27.4%). We also detected lower Drosha expression in the ectopic endometrium of women with adenomyosis than in the eutopic endometrium of the same women (59.6±3.2% *vs* 69.9±3.4%, respectively) (P=0.004; 95%CI of the difference: 2.3 to 16.7%). Additionally, we observed a moderate correlation between Drosha expression in the ectopic and eutopic endometrium of women with adenomyosis (P=0.034, rho=0.454).

No difference in Dicer expression was observed between the eutopic endometrium of women with adenomyosis and the eutopic endometrium of women without the disease (0.1±0.0% *vs* 2.4±1.8%, respectively) (P=0.399; 95%CI of the difference: -0.01 to 2.01%). We also did not find any difference in Dicer expression between the ectopic endometrium of women with adenomyosis and the eutopic endometrium of the same women (0.2±0.0% *vs* 0.1±0.0%, respectively) (P=0.218; 95%CI of difference: -0.3 to 0.05%).

We did not observe any difference in Exportin-5 expression between the eutopic endometrium of women with adenomyosis and the eutopic endometrium of women without the disease (76.3±2.6% *vs* 67.8±7.6%) (P=0.428; 95%CI of the difference: -18.1 to 7.9%). No difference in the expression of Exportin-5 was seen between the ectopic endometrium of women with adenomyosis and the eutopic endometrium of the same women (72.4±3.4% *vs* 76.3±2.6%) (P=0.337; 95%CI of the difference: -4.0 to 13.6%).

**Figure 1:**
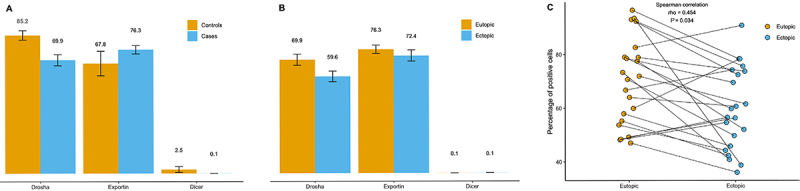
Expression of Drosha, Exportin, and Dicer in the endometrium of women with and without adenomyosis. **A**, Percentage of positive cells in eutopic endometrium from women without and with adenomyosis. **B**, Percentage of positive cells in eutopic and ectopic endometrium from women with adenomyosis. Data are reported as the mean and standard error. **C**, Correlation of the percentage of positive cells in eutopic and ectopic endometrium from women with adenomyosis.

**Figure 2:**
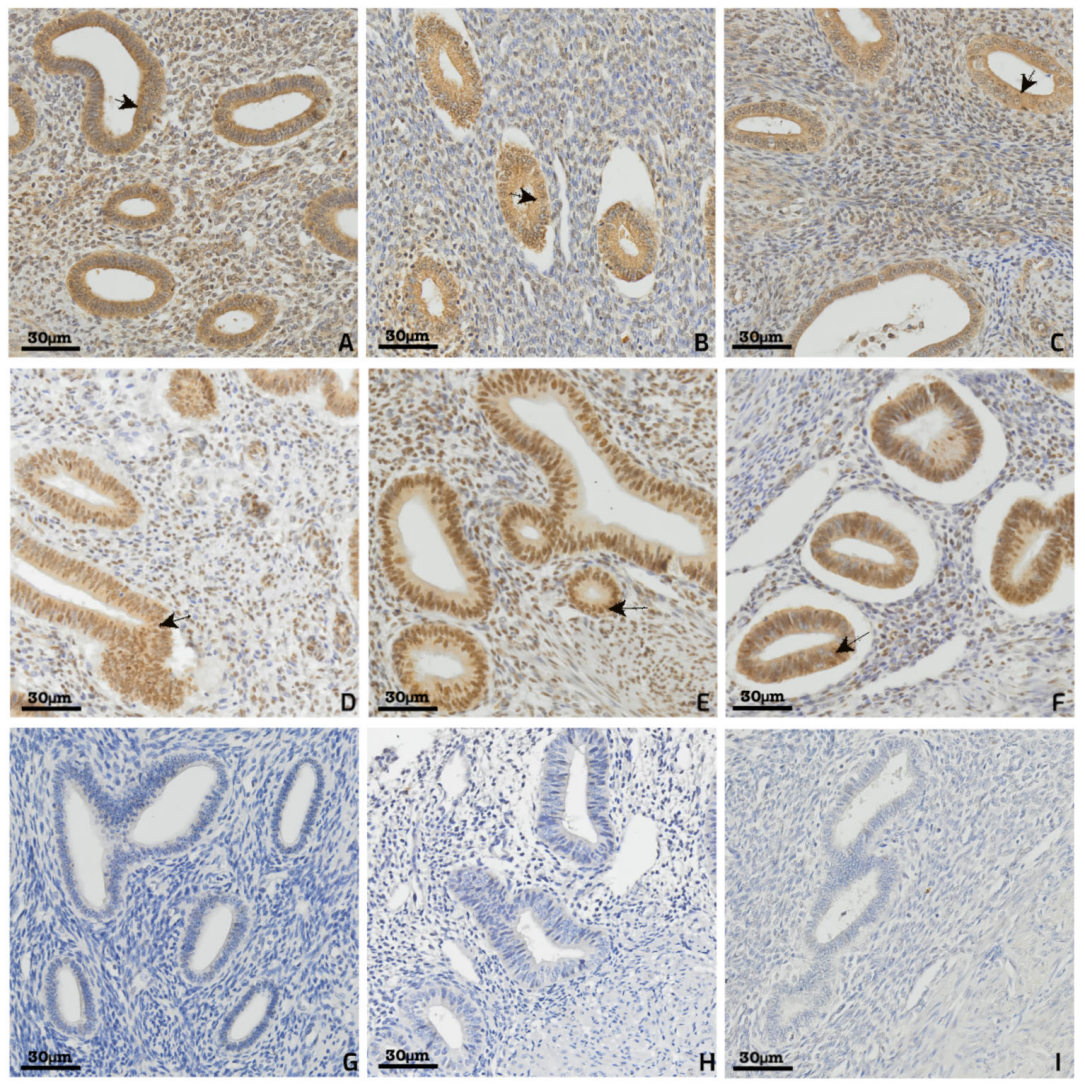
Immunohistochemical expression of Drosha, Exportin, and Dicer. Drosha in eutopic endometrium, without adenomyosis (**A**), in eutopic endometrium with adenomyosis (**B**), and in ectopic endometrium (**C**). Exportin in eutopic endometrium without adenomyosis (**D**), in eutopic endometrium with adenomyosis (**E**), and in ectopic endometrium (**F**). Dicer in eutopic endometrium without adenomyosis (**G**), in eutopic endometrium with adenomyosis (**H**), and in ectopic endometrium (**I**). Scale bars, 30 μm. Arrows represent luminal columnar epithelial cells that make up endometrial glands. A predominant pattern of cytoplasmic staining for the anti-Drosha antibody and both a nuclear and cytoplasmic pattern for the anti-Exportin antibody was observed.

Figure 1 graphically shows the results of the percent expression of proteins in tissues and Figure 2 shows the immunohistochemical labeling of antibodies in eutopic and ectopic endometrium. A predominant pattern of cytoplasmic staining for the anti-Drosha antibody and both a nuclear and a cytoplasmic pattern for the anti-Exportin antibody were observed.

## Discussion

Our pilot study showed that Drosha immunohistochemical expression is progressively lower in the eutopic and ectopic endometrium of women with adenomyosis compared to the eutopic endometrium of women without the disease. In addition, there was a significant correlation between protein expression in eutopic and ectopic endometrium of women with adenomyosis.

The reasons for the changes are unknown. It is not possible to say whether the detected changes were inherited or acquired by cells in some way throughout life. Recent studies have shown that these proteins play a relevant role in endometrial receptivity and endometrial cancer. In endometrial cancer, reduced Drosha expression was associated with poor clinical outcomes and reduced disease-specific survival, also justifying differences in miRNA expression profiles (24). Another interesting point is that Drosha has relevant non-canonical functions, such as maintaining genomic integrity as it is a key protein in the activation of the DNA damage response (DDR) in humans (26). The finding of KRAS somatic mutations in 40% of adenomyotic tissue cells suggests that adenomyosis harbors signs of genomic instability (14). In addition to pri-miRNA, other hairpin structures can also be targets of Drosha, such as some exons of mRNAs, and the level of such transcripts are upregulated after Drosha depletion (27). Furthermore, another important element that can arise from Drosha depletion is the loss of the microprocessor complex which, in turn, leads to the loss of stem cell properties and their premature differentiation into neural progenitors, interfering with neurogenesis (27), and these mechanisms are probably involved in the genesis and/or maintenance of adenomyosis (28). Based on these findings, although plausible, it is not possible to guarantee whether these common changes can explain the increased risk of endometrial cancer seen in women with adenomyosis (29). Another justification could be the involvement of sex steroid hormones in regulating the expression of the miRNA process. It is known that nuclear steroid hormone receptors, including ER-alpha, modulate miRNA biosynthesis through interference in the Drosha complex (30) and that the expression of this receptor isoform is expressed differently in the adenomyotic endometrium (31).

Dicer deletion can directly or indirectly cause depletion of progesterone receptors (32), whose lower expression could also be observed in adenomyosis (14). However, our data were not enough to prove a difference in Dicer expression, maybe even due to its low expression in the proliferative phase of the menstrual cycle.

From the genetic susceptibility point of view, some studies identified an association between Drosha and/or Dicer polymorphisms and the risk of endometriosis (33) and recurrent spontaneous abortion (34), conditions known to be associated with adenomyosis (35). Another hypothesis - although it is not safe to use it to explain the variation in the expression of these proteins in adenomyosis specimens - is the involvement of proteins p53, p63, and p73. These proteins can modulate Dicer and Drosha expression (36) and appear to be altered in adenomyosis specimens (37).

Our study had strengths and limitations. The selection of patients and controls was very rigorous; although necessary, strict criteria led to a significant reduction in eligibility. Nevertheless, we consider this point fundamental for a pilot study. Patterning the phase of the menstrual cycle also appears to be imperative, since sex steroids can drastically interfere with the expression of these proteins (38). In this study, we did not include samples from women using progestin, since we believe this could have interfered with the tissue expression analysis of target proteins. Furthermore, our sample did not contain samples from the secretory phase, as almost all hysterectomy surgeries were performed in the proliferative period of the menstrual cycle. Another point is the use of the immunohistochemical technique. However, although RNA sequencing (RNAseq) is considered the gold standard for gene profile evaluation at the transcription level, the assessment of protein expression by immunohistochemistry in tissue samples embedded in paraffin and fixed in formalin has shown high and statistically significant correlations with RNAseq (39). We also recognize that there are other excellent methods to identify and quantify protein expression such as the western blot, but in this pilot study we considered that immunohistochemistry would be of great benefit to detect the location of the target protein within the tissue sample. Additionally, the use of digital image analysis methodology with the supervision and checking by a pathologist adds substantial value to the quantitative measurement of immunohistochemical staining (40).

In conclusion, Drosha expression was significantly lower in the endometrium of women with adenomyosis compared to the eutopic endometrium of asymptomatic women without endometrial disease. Furthermore, although there was a significant correlation, protein expression was lower in the ectopic endometrium compared to the eutopic endometrium of women with adenomyosis. These findings, together with the evidence of the relationship between Drosha protein and endometrial cancer, indicate the direction of future research to understand the role of Drosha in the pathophysiology of adenomyosis and, eventually, its relationship with endometrial cancer.
